# Towards the Development of Artificial Bone Grafts: Combining Synthetic Biomineralisation with 3D Printing

**DOI:** 10.3390/jfb10010012

**Published:** 2019-02-20

**Authors:** Mima Kurian, Ross Stevens, Kathryn M McGrath

**Affiliations:** 1 The MacDiarmid Institute for Advanced Material and Nanotechnology, The School of Chemical and Physical Sciences, Victoria University of Wellington, Wellington 6012, New Zealand; mima.kurian@vuw.ac.nz; 2 The School of Architecture and Design, Victoria University of Wellington, Wellington 6012, New Zealand; ross.stevens@vuw.ac.nz; 3 The University of Technology Sydney, Ultimo NSW 2007, Australia

**Keywords:** Chitosan-calcium carbonate, Biomineralisation, Nacre, Bone, Biocomposites, 3D printing, hydrogel

## Abstract

A synthetic technique inspired by the biomineralisation process in nacre has been previously reported to be effective in replicating the nanostructural elements of nacre in 2D chitosan hydrogel films. Here we evaluate the applicability of this synthetic biomineralisation technique, herein called the McGrath method, in replicating the flat tabular morphology of calcium carbonate and other nanostructural elements obtained when 2D chitosan hydrogel films were used, on a 3D porous chitosan hydrogel-based scaffold, hence developing 3D chitosan-calcium carbonate composites. Nozzle extrusion-based 3D printing technology was used to develop 3D porous scaffolds using chitosan hydrogel as the printing ink in a custom-designed 3D printer. The rheology of the printing ink and print parameters were optimised in order to fabricate 3D cylindrical structures with a cubic lattice-based internal structure. The effects of various dehydration techniques, including air-drying, critical point-drying and freeze-drying, on the structural integrity of the as-printed scaffolds from the nano to macroscale, were evaluated. The final 3D composite materials were characterised using scanning electron microscopy, X-ray diffraction and energy dispersive X-ray spectroscopy. The study has shown that McGrath method can be used to develop chitosan-calcium carbonate composites wherein the mineral and matrix are in intimate association with each other at the nanoscale. This process can be successfully integrated with 3D printing technology to develop 3D compartmentalised polymer-mineral composites.

## 1. Introduction

Nacre is an inorganic-organic composite of calcium carbonate and various organic components including β-chitin, silk-like proteins, and acidic glycoproteins rich in aspartic acid. The nanostructure of nacre is described as the “brick-mortar” structure wherein single crystal platelets of calcium carbonate are interlaced by thin layers of the organic matrix [1]. Owing to the optical properties and biological significance of nacre, a number of approaches have been applied in order to mimic the elements of the nanostructure of nacre on a laboratory scale. For example, the principles of, supramolecular assembly, alternate soaking, Langmuir-Blodgett films, self-assembled multi-layered films, sequential deposition and freeze casting, have all been used [2,3,4,5,6,7,8]. We describe here the applicability of a recently established specialised mineralisation technique, the McGrath method, inspired by the biomineralisation in nacre, in developing 3D printed chitosan-calcium carbonate composites. Several studies have reported that the mechanical strength of nacre is comparable to that of natural bone despite the relatively high mineral content in nacre [9,10]. As such, the composite fabrication technique developed in this research is envisioned to provide a framework to future research into developing artificial bone grafts that are biocompatible; support cell proliferation and growth structurally, and are mechanically compatible to natural bone. That is, they have the mechanical and structural integrity that appropriately conform to the native tissue.

**Figure 1:**
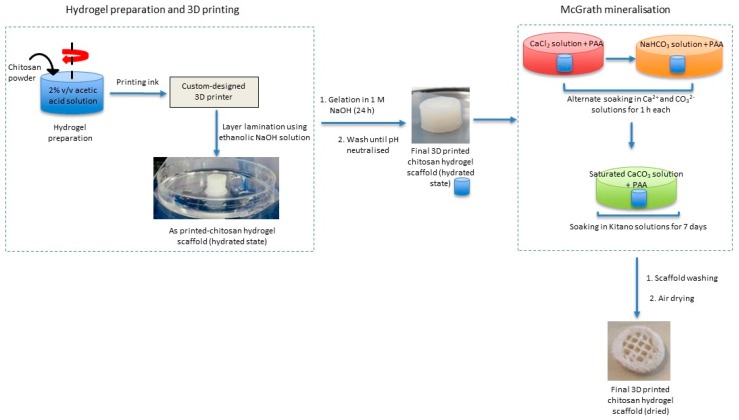
The experimental procedure, from left to right: preparation of the hydrogel printing ink and printing of the scaffold; gelation and neutralisation of the scaffolds; mineralisation.

This biomimetic mineralisation technique is combined with 3D printing technology to obtain a composite structure featuring chemical and structural characteristics of bone and nacre. Briefly, 3D chitosan hydrogel-based scaffolds with various structural features similar to natural bone (porous structure) are developed using a custom-designed nozzle extrusion-based 3D printer using chitosan hydrogel as the printing ink. These scaffolds are then mineralised via the McGrath method to obtain the desired composite (Figure 1).

3D printing allows for precise tailoring of the size, shape, interconnectivity, branching, geometry and orientation of the pores within a printed scaffold thereby enhancing control over its mechanical properties, biological effects and degradation kinetics [11,12]. This knowledge has more recently led to research into the application of 3D printing technology in the development of biologically relevant 3D structures towards tissue regeneration, in vitro organ development, bone fixtures and replacement materials, and anatomical models for preclinical drug screening [13,14,15]. Additive fabrication without the use of lasers, i.e., powder-based 3D printing was first developed at (MIT) Massachusetts Institute of Technology [16]. Laborious post-printing processing due to clogging of pores by unbound powder and the presence of organic solvents in the binders is a major drawback of this technology when it comes to biological applications. Hence, for prospective biological applications, extrusion-based 3D-bioplotting supplanted powder-based techniques. 3D-bioplotters use polymer hydrogel-based inks and do not rely on high temperatures in order to fabricate stable 3D scaffolds [17]. An extrusion-based 3D printer custom-designed with the capability of using hydrogels as the printing ink is used to develop 3D scaffolds for this study.

The first report on 3D printed chitosan hydrogel-based scaffolds was in 2002, which indicated good in vitro biocompatibility and enhanced osteoblast cell proliferation in symmetric lattice-based chitosan-hydroxyapatite composite scaffolds [18]. Geng et al. demonstrated the feasibility of using chitosan hydrogel-based inks in a dual extrusion robotic printer to print irregularly shaped cranial patches based on computed tomography scan images of the region that were converted to printer compatible models [11]. Furthermore, Lim et al. experimented with the application of cryogenic rapid prototyping by using a cold printing platform to freeze the printed structures during printing in order to develop chitosan hydrogel-based scaffolds with controlled microarchitecture (pore size and pore orientation). They found that the scaffold architecture affected both cellular infiltration and neo-vascularisation [19]. More recently, Lee et al. found that 3D scaffolds developed via this method exhibited very low mechanical strength and thus were not applicable as load-bearing bone grafts. Nonetheless, these may be considered for soft tissue regeneration applications [20]. Almeida et al. studied the in vitro inflammatory responses of 3D printed chitosan scaffolds with different compartmental geometries and showed that these scaffolds promote adhesion and proliferation of monocytes and the mobility of macrophages. They also found that varying the surface geometry resulted in changing the proliferating-cell morphology and hence their inflammatory responses [21].

The McGrath mineralisation method was found to be an effective technique in developing calcium carbonate formations similar to those observed in nacre biomineralization in laboratory conditions on both carbohydrate-based and protein-based thin films [22,23]. The method involves soaking a porous wet biopolymer hydrogel thin film alternately in reactive mineral precursor ion solutions and then soaking the now preloaded scaffold in a saturated solution of calcium carbonate for a prolonged period. In the presence of polyacrylic acid (PAA), this mineralisation method results in the formation of uniformly distributed pancake-like CaCO_3_ crystallites throughout the thin film [24]. This morphology of CaCO_3_ crystallites is similar to what is observed during the early stages of growth during the nacre biomineralisation process [25]. Here, we investigate the prospect of using the McGrath method to achieve a similar mineral formation within the layered structure of 3D printed chitosan hydrogel-based scaffolds.

The prospect of fabricating structurally porous 3D scaffolds using chitosan hydrogel as the printing ink in a custom-designed nozzle extrusion-based 3D printer employing only physical crosslinking chemistry was investigated. The rheology of the printing ink and print parameters were optimised to fabricate 3D porous structures. The structural integrity of the scaffolds was explored using electron microscopy following dehydration of the scaffolds (required due to the inclusion of a dehydration step in the mineralisation protocol for the 2D chitosan thin films [22], on which the mineralisation used here is based) and subsequent rehydration. The final dehydrated inorganic-organic composites developed by mineralisation of the 3D printed scaffolds were characterised via scanning electron microscopy (SEM), energy dispersive spectroscopy (EDS) and X-ray diffraction (XRD).

## 2. Results

### 2.1. Preparation of the Printed Scaffolds

Previously, Munro et al. had used 2% *w*/*v* chitosan hydrogel prepared in the presence of 1% *v*/*v* acetic acid solution to develop 2D thin films which were then mineralised to obtain nacre-like calcium carbonate formation [22]. However, when using the same solution for fabricating 3D scaffolds, it was observed that the viscosity of the chitosan hydrogel at this concentration was too low, making it unsuitable to be used as the printing ink in a nozzle extrusion-based 3D printer. For a hydrogel to be deemed a suitable printing ink, it is essential for it to show certain rheological properties including shear thinning when subjected to increasing shear strain and shape retention upon strain cessation [26]. Hydrogels with favourable flow properties can be (1) extruded through the nozzle as a continuous strand and then (2) rapidly cease to flow after extrusion thus resulting in a stable structure. By monitoring the rheological properties and extrusion behaviour of chitosan hydrogels of different concentrations, 5% *w*/*v* chitosan hydrogels prepared in 2% *v*/*v* acetic acid (the higher concentration of acetic acid was required in order to improve the solubility of the chitosan) were selected as the printing ink.

**Figure 2:**
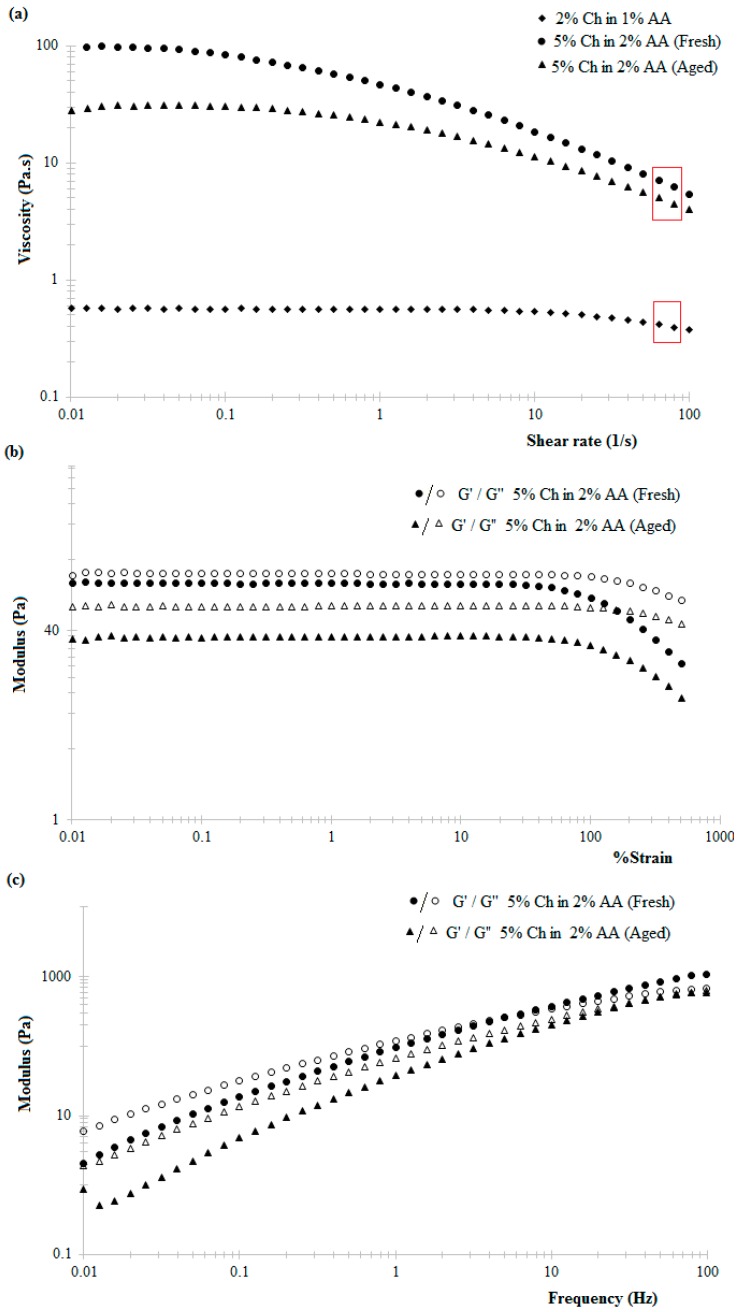
The log-log plots for the variation in (**a**) viscosity with increasing shear rate; (**b**) the storage (G′) and loss (G″) moduli as a function of strain (1 Hz, strain sweep); (**c**) G″ and G′ as a function of frequency, 2% strain (frequency sweep), for freshly prepared and aged 5% *w*/*v* chitosan hydrogel (Ch) prepared in 2% *v*/*v* acetic acid (AA). The estimated shear rate experienced at the tip of the printing nozzle is highlighted by the red boxes.

In Figure 2a exemplary flow curves for chitosan hydrogels at two different chitosan concentrations are shown. From Figure 2a, it can be identified that the viscosity of a 5% *w*/*v* chitosan hydrogel prepared in the presence of 2% *v*/*v* acetic acid at low shear rates is higher than that of 2% *w*/*v* chitosan hydrogel prepared in the presence of 1% *v*/*v* acetic acid. Shear-thinning behaviour was observed in the case of both the hydrogel formulations. In the case of the 2% *w*/*v* chitosan hydrogel, shear thinning was observed at slightly higher shear rates than in the case of 5% *w*/*v* chitosan hydrogel. In addition, there was significantly less variation in the viscosity of the former at lower shear rates, with the hydrogel essentially behaving like a Newtonian fluid. The estimated shear rate at the tip of the 0.42 mm internal diameter nozzle, when printing at a speed of 6 mm s^−1^, was 72 ± 7 s^−1^. Comparing the flow behaviour of the two hydrogels at this shear rate (marked by the red box in Figure 2a) showed that the viscosity of 5% *w*/*v* chitosan hydrogel in that region is much higher than that of 2% *w*/*v* hydrogel, suggesting that the former may have better continuity when extruded, a critical property that attributed for the hydrogel to be used as the printing ink in the extrusion-based 3D printer.

Trial 3D printing was undertaken using the chitosan hydrogels of different concentrations confirming the results obtained from the rheology study. As the chitosan concentration was increased more continuous strand formation and greater shape retention were observed. If the chitosan concentration was too high, the required pressure to induce flow and achieve an appropriate viscosity was beyond the range available in the printer used here. 5% *w*/*v* chitosan hydrogels prepared in the presence of 2% *v*/*v* acetic acid was found to have rheological properties suitable for use as a printing ink in the custom-designed 3D printer used in this study. All 3D scaffolds described herein were developed using this hydrogel ink.

The variation of the rheological properties of 5% hydrogel was also explored as a function of ageing to determine if printing inks could be prepared ahead of time or if only freshly prepared hydrogels could be used. In Figure 2a are shown the flow curves for a freshly prepared 5% *w*/*v* chitosan hydrogel and a 6-day aged sample as a function of increasing shear rate. The data suggest that the viscosity of chitosan hydrogels decreases upon ageing. This observation is consistent with various literature reports owing this behaviour to polymer degradation and subsequent changes in macrochain conformations and gel structure [27,28]. The lowering of the viscosity upon storing may also be due to slow protonation of the amine group with time resulting in more liquid-like behaviour [29]. Such variations will affect the extrusion behaviour of the hydrogel [30].

The variations observed in response to changes in strain and frequency of fresh and aged 5% chitosan hydrogel are shown in Figure 2b,c. The data indicate the following: (a) the viscosity versus shear rate plots show that aged and freshly prepared hydrogels have similar flow behaviour (shear thinning upon increasing shear rate); and that the viscosity, at shear rates experienced at the tip of the nozzle during printing, are comparable; (b) the strain sweep data indicate that the storage modulus of the freshly prepared hydrogel is higher than the aged samples; while (c) the frequency sweep (Figure 2c) indicates that both aged and freshly prepared hydrogels undergo a transition from primarily viscous (G′ > G″ at lower frequencies) behaviour to primarily elastic behaviour (G′ > G″ higher frequencies) upon increasing frequency. The crossover-point, where G′ = G″, increased as the gels aged. In the case of freshly prepared hydrogels, the crossover occurred at approximately 6.3 Hz compared to 50 Hz observed in a 6-day aged sample. On comparing the printability of fresh versus aged gels, it was observed that the former had better shape retention after extrusion. Practically, it was found that stable prints were produced when using freshly prepared hydrogel or within 3 days of preparation. This conforms with the rheology data, where the viscosity of the gels decreases upon ageing due to ongoing structural changes in the gels, thus confirming that there is a range of gel viscosities that is optimal for 3D printing. As such, all other things being equal it is concluded that hydrogels with lower crossover frequencies (nearby 6 Hz) and hence better shape retention are a better choice as a printing ink when using an extrusion-based 3D printer as described here.

### 2.2. Characterisation

**Figure 3:**
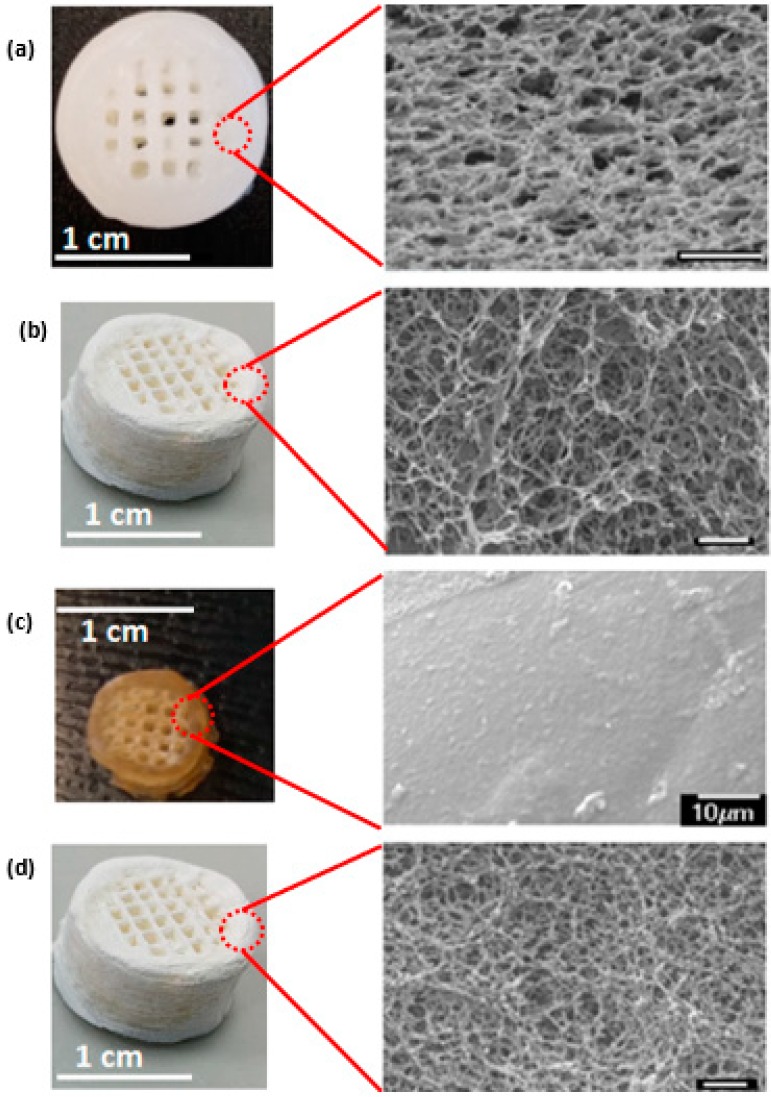
On the left are optical images of non-mineralised 3D chitosan hydrogel-based scaffolds in hydrated or dehydrated states. On the right are corresponding electron microscope images. 3D chitosan hydrogel-based scaffold in (**a**) hydrated; (**b**) critical point-dried; (**c**) air-dried; (**d**) freeze-dried. Scale bar is 1 μm unless otherwise mentioned.

Cryo-SEM images of the as-fabricated chitosan hydrogel scaffolds show that the hydrogel matrix is inherently highly porous with an interconnected network of nano to microscale pores in addition to the macropores incorporated in the computer-aided design (CAD) model (Figure 3). This variation of porosity imparts within the final 3D printed scaffolds hierarchal porosity when in a hydrated state. The McGrath mineralisation method, as described by Munro et al. [22] includes a dehydration step prior to the alternate soaking step. Hence it was necessary to explore the structural integrity of the 3D chitosan scaffolds following dehydration. Exploring the structural integrity of the scaffolds also yielded some initial crude information on their mechanical strength. We note that the mechanical behaviour of the chitosan scaffolds and final composites was extensively investigated and will be reported in a separate manuscript.

SEM images showed that the degree of retention of the micro and nanoscale pores in dehydrated scaffolds varied depending on the method of dehydration. Pore collapse due to surface tension effects of the evaporating solvent during the dehydration process was observed. In Figure 3 optical and SEM images of hydrated 3D chitosan hydrogel-based scaffolds are compared with those of dehydrated scaffolds obtained using the three different dehydration methods. The SEM images (Figure 3b,d) of the freeze-dried and the critical point-dried scaffolds reveal that the porous network is essentially retained and is externally accessible after drying with negligible gross dimensional shrinkage in the 3D structure. Hence the structural integrity of the scaffolds in maintained. However, in the case of the former, a thick non-porous surface skin is present in the regions of the scaffold in contact with liquid nitrogen, which is a common artefact observed when freeze-drying hydrogels [31]. Air- drying, on the other hand, resulted in more than 50% shrinkage in the gross dimensions of the as-fabricated scaffolds on a macroscale and at a microscale, no micro or nanoscale pores were observed.

Simple handling of the variously dried samples using tweezers indicated striking differences in their mechanical responses upon compression. It was observed that both freeze-dried and critical point-dried scaffolds exhibited primarily foam-like behaviour i.e., the samples deformed irreversibly. Air-dried scaffolds, in contrast, did not show any deformation under the same conditions.

The swelling behaviour of the scaffolds dehydrated using each of the three methods was studied under an in vivo-like environment (similar pH conditions) (see Table 1). This provided further information on the structural integrity of the scaffolds. Rehydration of the dried scaffolds in 1× PBS showed a negligible increase in the volume of the freeze-dried and critical point-dried scaffolds. Irrespective of the extent of hydration, these samples retained their macroscopic volume and shape. Following rehydration, these samples did not display the compression response seen in the as-printed hydrogel scaffolds. That is, while they retained their 3D structure the changed state of the chitosan chains meant that the samples retained their limited resistance to compression that was characteristic in their dehydrated states.

**Figure 4:**
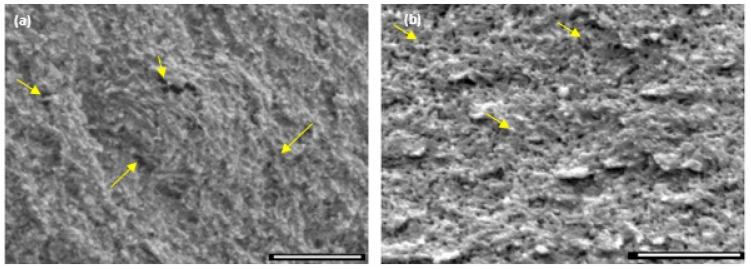
Cryo-scanning electron microscopy (SEM) images of air-dried chitosan hydrogel-based scaffolds (**a**) before and (**b**) after soaking in 1× PBS for 1 week. The yellow arrows highlight some of the nano/microscale pores within the region of observation. The scale bar is 1 μm.

In the case of air-dried scaffolds, although the swelling ratio was lower, the increase in the volume of the scaffolds suggests an increase in the porosity upon rehydration. This was confirmed using cryo-SEM analysis of rehydrated air-dried scaffolds (Figure 4). Here the presence of a large number of nano and microscale pores are revealed in the hydrogel matrix in the rehydrated state in comparison to the corresponding dehydrated state. The regeneration of the hierarchical porosity with length scales from the nano to macro level is facilitated by diffusion dependent swelling of the scaffold combined with an inherent connectivity of the pores through an open network. An increase in the volume of the scaffold was observed only in the first 24 h of soaking in PBS, no further changes were observed with longer soaking times. Furthermore, the enhanced resistance to compression was maintained.

These results show that air drying enables the porosity to be readily and reversibly varied upon hydration and dehydration, while also enabling the resistance to compression to be increased. This may be advantageous for in vivo applications.

### 2.3. Mineralisation

Having achieved good printing conditions and initially ascertained in gross both the state of porosity, in terms of size range and connectivity in hydrated and dehydrated scaffolds, the next phase in developing the final composite materials was to mineralise the scaffolds. As previously stated, the method followed for mineralisation of 2D chitosan hydrogel scaffolds involves oven-drying the wet hydrogel films at 37 °C and then soaking in 1 M NaOH to achieve enhanced gelation prior to being exposed to the McGrath mineralisation process [22]. However as noted above, air-drying results in considerable shrinkage of the 3D printed scaffolds and the collapse of nano and micro-sized pores. Such collapse significantly reduces diffusion of the mineralisation solutions throughout the sample, which is otherwise straightforwardly achieved in the fully hydrated state. Thus, it was deduced that maintaining the dehydration step in the mineralisation process would reduce the extent of diffusion. Thus, the mineralisation method was modified. The oven-drying process was eliminated and the scaffolds were mineralised while in the hydrated as-printed state. Through this modified method, it is expected that the extent of mineralisation achieved within the 3D scaffolds would be increased while also facilitating enhanced interaction between the mineral ions and the polycationic chitosan (in a hydrated state).

**Figure 5:**
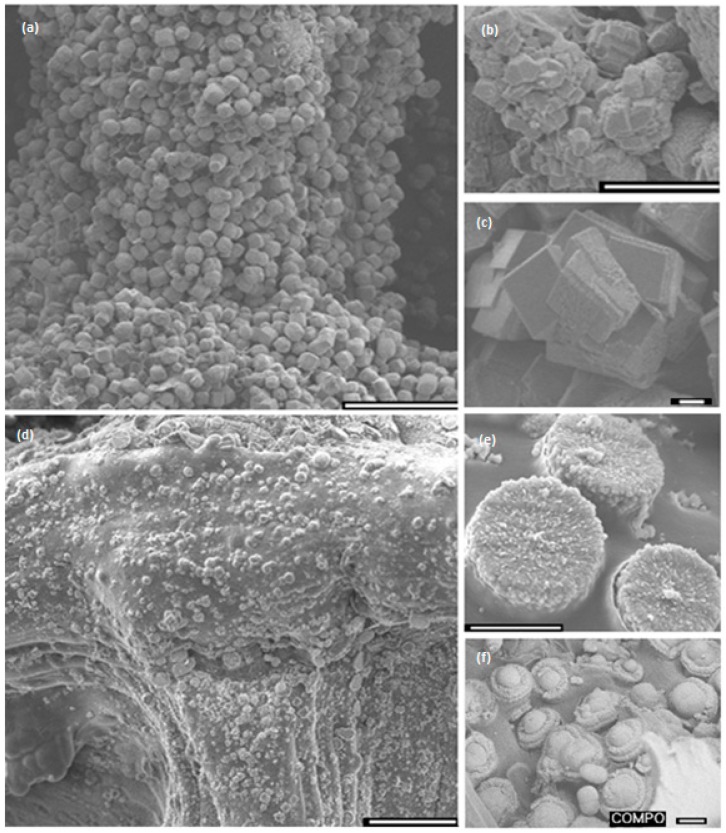
SEM images of 3D chitosan hydrogel-based scaffold mineralised via the McGrath method (**a**–**c**) in the absence of polyacrylic acid (PAA), and (**d**–**f**) in the presence of 2.5% *w*/*w* PAA with respect to the dry weight of the chitosan scaffold used; (**e**) pancake-like CaCO_3_ crystallites (**f**) pancake-like crystallites with globular moieties growing on the surface. Scale bar for images on the left-hand side is 100 μm and for those on the right-hand side is 10 μm.

Scaffolds were mineralised when in their hydrated state via a combination of alternately pre-soaking the scaffolds in the precursor ion solutions followed by exposure to a saturated calcium carbonate solution (Kitano solution)–together herein called the McGrath method. SEM images of the resulting composites are shown in Figure 5 (alternate soaking in 0.1–0.5 M mineral precursor ion solutions for 24 h each followed by 7 days of soaking in Kitano solution). The mineralised scaffolds showed extensive CaCO_3_ formation in the regions of the scaffold directly exposed to the mineralisation media and in between the printed layers (Figure 5a–c). The resultant crystallites exhibit rhombohedral, spherical, rosette or stacked platelet-like morphologies.

When PAA was added to each of the mineralisation solutions (2.5% *w*/*w* with respect to the dry weight of the 3D chitosan scaffold used), the morphology of the resultant calcium carbonate crystallites varied from the traditional structures obtained in the absence of PAA. The morphology, in this case, varied depending on the mineralisation conditions (concentration of mineralisation media used and the period of exposure to the mineralisation media) used. The morphology varied from pancake-like CaCO_3_ crystallites to globular structures growing on top of pancake-like structures (Figure 5d–f) with the increase in the concentration of the precursor ions used from 0.1 M to 0.5 M. The pancake-like morphology obtained when using 0.1 M precursor ion solutions, is similar to that obtained when 2D chitosan hydrogel thin films were used as the mineralisation scaffold [22]. In similarity to when scaffolds were mineralised in the absence of PAA, extensive mineralisation was observed in the regions of the scaffold exposed to the mineralisation media and within the print layers.

**Figure 6:**
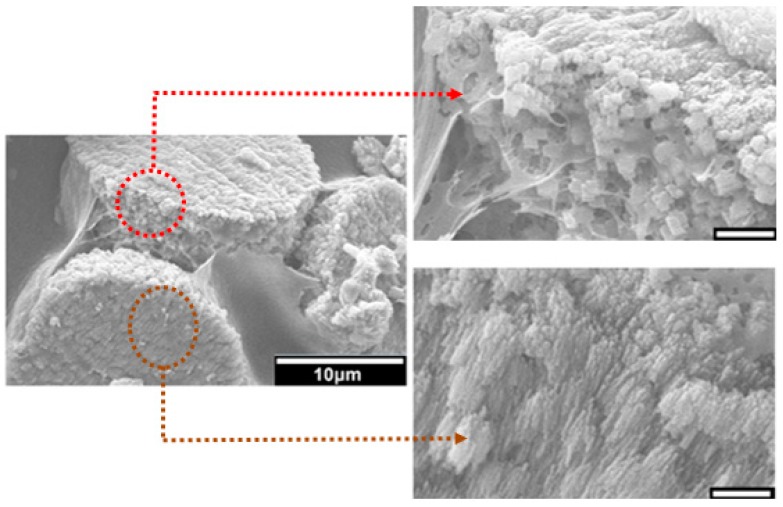
SEM images of the top surface and lateral view of pancake-like calcium carbonate crystallites formed during the mineralisation of 3D chitosan hydrogel-based scaffolds via the McGrath method in the presence of 2.5% *w*/*w* PAA (with respect to the dry weight of the chitosan scaffold used). The scale bar is 1 μm unless otherwise stated. Note the orientation of the nanocrystals relative to the organic scaffold.

Furthermore, in the case of composites developed by mineralisation in the presence of PAA, at a higher magnification, it is observed that individual pancake-like crystallites are formed of oriented aggregates of numerous CaCO_3_ nanoplatelets. These aggregates are embedded within the chitosan polymeric matrix (Figure 6). This kind of physical association between the organic matrix and the mineral component is observed only when PAA is incorporated in the mineralisation method. Again this conforms with the results obtained when using 2D chitosan thin films [32].

**Figure 7:**
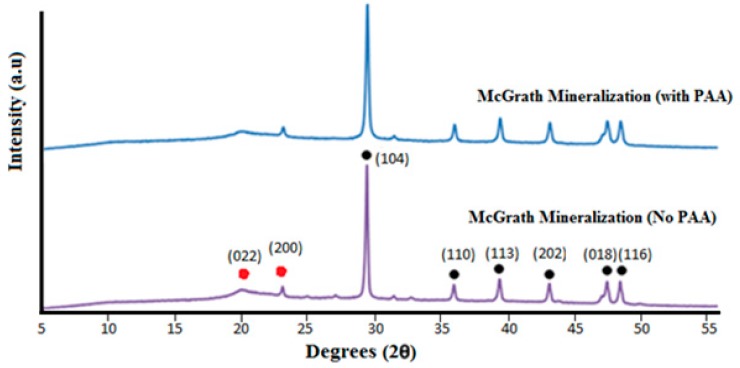
X-ray diffraction (XRD) plots for scaffolds mineralised via the McGrath method in the presence or absence of PAA. In both cases, 2.5% *w*/*w* PAA (with respect to the dry weight of the chitosan scaffold used) was present in all mineralisation solutions. The black and red dots represent the prominent 2θ peaks corresponding to calcite and crystalline regions within the chitosan scaffold respectively.

Powder XRD analysis shows that the CaCO_3_ predominantly exists as calcite in the absence or presence of PAA based on the prominent peak observed at 2Ɵ = 29.4° corresponding to the (104) plane. The additional peaks observed at 2Ɵ = 10.2° (020), 19.8° (022) and 22.0° (200) correspond to crystalline sites within the chitosan polymer matrix (Figure 7) [32].

**Figure 8:**
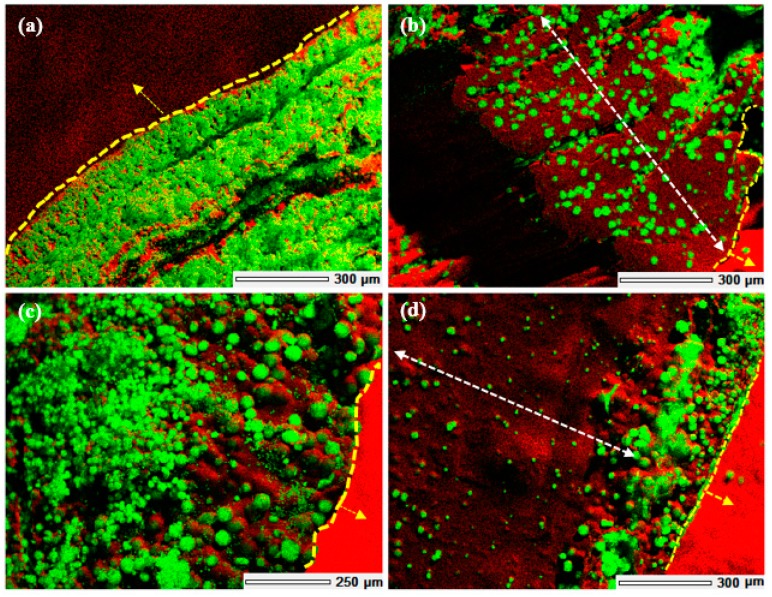
Energy dispersive spectroscopy (EDS) images of McGrath mineralised (in the presence of 2.5% PAA (with respect to the dry weight of the chitosan scaffold used) chitosan hydrogel-based scaffolds fabricated via (**a**,**b**) 3D printing and (**c**,**d**) bulk gelation. (**a**,**c**) Show the external surface of the scaffolds directly in contact with the mineralisation solution and (**b**,**d**) show the transverse cross-section of the scaffolds (i.e., the region not exposed directly to the mineralisation solution). Calcium is shown in green and carbon in red. The regions marked by the white arrows represent those that were not exposed to the mineralisation solution directly. The regions marked by yellow dashes and arrows represent the external surfaces that are not a part of the scaffold.

When comparing the mineral formation in 3D scaffolds fabricated via 3D printing and bulk gelation, it was observed that extensive mineralization was achieved in the case of the former. EDS images of the cross-section (along the *z*-axis) of composites fabricated by either of the methods and mineralised via the McGrath method (in the presence of 2.5% *w*/*w* PAA with respect to dry weight of the chitosan scaffold used) are shown in Figure 8. EDS images confirm that the extent of mineral formation (in the interior and the exterior regions of the 3D scaffold) is higher in the case of the 3D printed scaffolds compared to the 3D scaffolds generated via bulk gelation without any macropores. This indicates that mineralisation throughout the scaffolds is facilitated by the presence of macropores and print layers achieved by 3D printing of the scaffolds.

## 3. Discussion

Since the first reported investigation in 2002 by Ang et al. where 3D printing was used to generate chitosan hydrogel scaffolds, there has been considerable research focused on developing porous scaffolds with tailored porosity and pore interconnectivity [33,34]. However, most of these studies reported 3D chitosan scaffolds in the form of in vivo implants that resulted in the formation of a bone matrix at the implant site or as 3D composites developed using blended chitosan-mineral hydrogels (hydroxyapatite/tricalcium phosphate) as the printing ink [34,35,36]. Although these reported techniques were effective in the fabrication of 3D chitosan hydrogel-based scaffolds with controllable porosity, pore interconnectivity, osteoconductivity and bone mineralisation; the mechanical strength was not optimised to a range suitable for in vivo load-bearing bone-graft applications [37].

The mechanical strength of a biopolymer-composite can be improved by manipulating various aspects of the material including its porosity, scaffold architecture, polymer density, mineral content and the morphology of the mineral crystallites that act as fillers within the polymer matrix [16,31]. In this work, 3D printing is used as a technique towards developing regularly porous chitosan hydrogel-based scaffolds and the McGrath mineralisation method is used as a biomimetic method to incorporate minerals into the polymer matrix ensuring strong mineral-matrix interactions. The latter is envisioned to improve the mechanical properties of the final composite structure despite the porous architecture (as noted above the mechanical characteristics of the composites will be reported in a separate article).

Determination of the critical control parameters within the experimental protocol was undertaken. The relevant data for each experimental parameter are discussed below starting with the viscoelastic behaviour of the printing ink; the chitosan hydrogel required to produce stable scaffolds conforming to the original CAD pattern.

### 3.1. Fabrication of 3D Printed Chitosan Hydrogel-Based Scaffolds

The observed shear-thinning behaviour of the chitosan hydrogels within increasing shear rate is attributed to the tendency of the applied shear energy to align anisotropic molecules and untangle polymeric chains or large aggregates. This reduces the hydrodynamic drag and consequently reduces the energy dissipation and increasing tendency of the material to flow [38]. Literature suggests that chitosan hydrogels exhibit shear-thinning behaviour under low shear rates at 25 °C, this behaviour being more prominent in the case of higher concentration hydrogels, similar to the data reported here (Figure 2a) [28]. Similarly, the decrease in the viscosity of 5% *w*/*v* chitosan hydrogel in 2% *v*/*v* acetic acid solution with time (Figure 2a) can be attributed to the slow dissolution via protonation of the amine groups of the chitosan polymer chain when in the presence of acetic acid [39].

**Figure 9:**
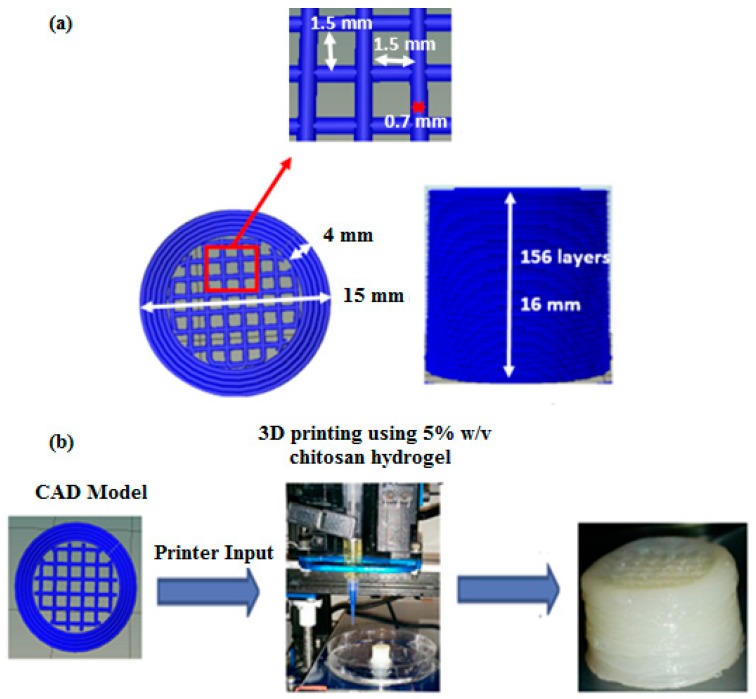
(**a**) Specifications of the computer-aided design (CAD) model input for the 3D printer and (**b**) images from the experimental procedure.

A further important consideration when using 3D printing to produce scaffolds is the strength of the adhesion between the printed layers, resulting in good structural integrity including resistance to delamination and layer slippage. Using 5% *w*/*v* chitosan hydrogel prepared in the presence of 2% *v*/*v* acetic acid, structurally stable 3D scaffolds were fabricated (refer to the optical image of the 3D scaffold in Figure 9). The layers of printed hydrogels were laminated to each other; the adhesion is strong enough to avoid layer slippage hence forming a stable 3D structure. Ethanolic sodium hydroxide, used to laminate the printed layers to each other, partially deprotonates the amine groups in the printed hydrogel layers such that printed layers do not flow and adhere to each other with appreciable adhesion. The concentration of the ethanolic sodium hydroxide was optimized to 0.7% *v*/*v*–1.5% *v*/*v* (30:70 ethanol:NaOH solution) such that printed layers do not flow and collapse at the same time ensuring that the layers are not completely deprotonated in which case the printed layers will not adhere to each other.

### 3.2. Extent of Scaffold Hydration

The response of the scaffolds to different environmental conditions, particularly the extent of hydration is very important in defining their potential biomedical applications. Critical point-drying and freeze-drying retain the porous network within the scaffold (Figure 3, nano through to micropores remain visible) making them suitable for biomedical applications. However, the inability of the dehydrated scaffolds to withstand deformation under compression (as exerted when compressing the scaffolds between tweezer tips) render them inapplicable for load-bearing bone graft applications. Such scaffolds may, however, be perused for soft tissue engineering applications.

Air-drying was selected as the most suitable method to dehydrate the as-printed scaffolds (both mineralised and non-mineralised scaffolds), of the three methods investigated, despite the observed dimensional shrinkage, since air-dried scaffolds were found to be structurally stronger, depicting good resistance to deformations under similar stress conditions as above. The dimensional shrinkage can be attributed to the closure of the micro and nanoscale pores due to the surface tension effects of the evaporating water during air-drying. The collapse of the micro and nanoscale pores results in the compact stacking of the printed layers within the final structure and thus offers a higher resistance to deformation under compressive stress compared to the highly porous counterparts.

The rehydration behaviour of dehydrated scaffolds was explored considering the relevance of a hydrated environment in in vivo conditions and to analyse their ability to regain the lost microscale porosity. While the superior compressive behaviour of the air-dried scaffolds was deemed to be a sought-after distinguishing factor over the other two dehydration methods, the resultant loss of microscale porosity in the process potentially restricts the applicability of these scaffolds in vivo, wherein long-range porosity is often linked to nutrient exchange and the possibility of promoting vasculature through these structures.

The swelling behaviour of hydrogels is defined by the restricted solubility of polymer chains. This depends on the hydrogel structure (porous or non-porous), dehydration method used, polymer network density, crosslink density, solvent-polymer interactions, the pH and nature of the solvent [40,41,42]. Omidian et al. also explain swelling as a polymer chain dissolution-dependant process; since solvent diffusion into the dried polymer continues with time [43]. Herein, swelling observed in dehydrated 3D chitosan hydrogel-based scaffolds is expressed as the swelling ratio which is dependent on the solvent absorbance and the resultant change in the volume of the scaffolds. The swelling ratio is defined as the ratio of the weight of rehydrated scaffolds (due to solvent absorption) to that of dehydrated scaffolds. The percentage volume change, on the other hand, is related to the dimensional change observed in 3D scaffolds as a result of solvent absorbance.

The observation that the freeze-dried and critical point-dried scaffolds showed a higher swelling ratio in comparison to air-dried scaffolds (~5), despite no physical change in the scaffold volume, could be attributed to the presence of large number of nano and microscale pores within these structures that facilitate capillary action dependent solvent diffusion into the scaffold. Freeze-drying, however, renders the chitosan polymer chains crystalline, thus inhibiting any chain dissolution dependent swelling and subsequent change in the dimensions of the structure [17]. Although rehydration resulted in some of the lost porosity being regained in air-dried chitosan hydrogel-based scaffolds, the lower swelling ratio suggests that the total porosity of the rehydrated scaffolds was comparably lower than that of the scaffolds dried via the other techniques.

In the case of air-dried scaffolds, the lack of a porous network results in a lower swelling ratio compared to the others. However, the small increase in volume and the appearance of a large number of nanopores in the rehydrated scaffolds suggests that this technique does not render the polymer chains completely crystalline which would have inhibited any dissolution dependent change polymer swelling as in the case of freeze-dried samples. The increased porosity of rehydrated air-dried scaffolds together with the printed macropores could, therefore, contribute to biologically relevant porosity during in vivo applications [44,45]. These results together with the observation that air-drying can potentially reduce the susceptibility of 3D chitosan hydrogel-based scaffolds to deformation under compression are the main reason for preferentially selecting this dehydration technique.

### 3.3. Mineralisation of 3D Scaffolds

The formation of calcium carbonate throughout the 3D chitosan scaffolds, when mineralised via the McGrath method, is a result of heterogeneous nucleation of CaCO_3_ that is facilitated by the increase in pH resulting from the slow evolution of CO_2_ gas from the mineralisation solutions with time [22]. The extent of mineralisation throughout the scaffold is a diffusion dependent process enhanced by the presence of the interconnected porous network within the hydrogel matrix. The presence of printed macropores and the layer-by-layer build of the 3D scaffolds increases the effective surface area available for diffusion of the mineralisation media thus, enhancing the extent of mineralisation achieved in comparison to scaffolds fabricated via bulk gelation without any macropores or a layered structure.

Crystal growth modifiers such as PAA are known to modify the morphology of CaCO_3_ crystallites precipitated from a solution in the presence of a hydrogel matrix. It is also postulated that these can suppress crystal nucleation in solution, hence facilitating crystal growth in association with the matrix [46,47]. Herein, mineralisation of 3D chitosan hydrogel-based scaffolds the presence of PAA (Figure 5d–f) and low concentration of mineral precursor ions solutions (0.1 M) resulted in the formation of laterally growing pancake-like structures which are similar to the CaCO_3_ morphologies found in the early stages of nacre biomineralisation [25]. In the presence of PAA, literature suggests that, calcium carbonate nucleation and growth on chitosan hydrogels is directed by the 3D structural motif formed as a result of the electrostatic interaction between the COO^−^ groups in PAA and the NH_3_^+^ groups in chitosan, typically promoting lateral growth of the crystallites with respect to the hydrogel matrix [48]. The observed intimate association between the mineral and the matrix (Figure 6) suggest that the formation of a polyelectrolyte complex ensures nucleation and hierarchical growth of the resultant calcium carbonate crystallites from nano to microscale.

The variation in the morphology and distribution of CaCO_3_ crystallites throughout the 3D structure, without any variation in the polymorphic form, may be attributed to diffusion dependent variation in the concentration of the Ca^2+^ and CO_3_^2−^ ions across the scaffold and the relative extent of interaction between the mineral ions and PAA. Further research is required to optimise the morphology and distribution of the mineral throughout the 3D scaffolds. Additional factors to consider are the concentration of the precursor ions, the concentration of PAA used and the period of exposure to the mineralisation media.

## 4. Materials and Methods 

### 4.1. Materials

Analytical grade chitosan (MW: 100,000–300,000 g/mol), calcium chloride dihydrate (CaCl_2_·2H_2_O), and polyacrylic acid (MW: average 1800 g/mol) were purchased from Sigma Aldrich (Auckland, New Zealand). Acetic acid (Univar, Summit, IL, USA, AR grade), CaCO_3_ (Univar, AR grade), and sodium hydrogen carbonate (NaHCO_3_) (Panreac, Darmstadt, Germany) were used as received. All solutions used in this work were prepared using purified water (Sartorius Arium 611UV purification system (Goettingen, Germany), (18.2 MΩ cm resistivity)). 1X phosphate buffered saline (PBS) is prepared by dissolving 8 g NaCl (Pure Science Limited, Porirua, Wellington, New Zealand), 0.2 g KCl (Sigma Aldrich, Auckland, New Zealand), 0.24 g KH_2_PO_4_ (Mallinckrodt Barker Inc., Phillipsburg, NJ, USA) in 800 mL of purified water, after the pH is adjusted to 7.4 with HCl, a further 200 mL purified water is added.

### 4.2. 3D Printer Parameters

A custom-designed 3D printer equipped with syringe extruders, based on the open-source MendelMax 2.0 by Maker’s Tool Works was used (Figure S1, Supplementary Materials). Printing was performed at room temperature at a speed of 6 mm s^−1^ (speed of movement of printing tip) using a 5 mL Luer lock syringe attached with plastic nozzles. The rate of extrusion (feed rate, controlled as the pressure of extrusion) of the hydrogel is automatically controlled by the Slic3r software based on the input speed of printing and the diameter of the nozzle.

### 4.3. 3D Model 

3D Tinkercad, a browser-based computer-aided design (CAD) and modelling tool was used to design the preferred architecture of the scaffold to be printed. Repetier-Host V1.6.2 developed by Hot-World GmbH & Co.KG (Willich, Germany) was used as the CAD input interphase for the 3D printer.

The 3D scaffolds were designed to facilitate interconnected porosity while also imparting some mechanical strength. The specific CAD model comprising of a hollow cylinder enclosing cubic lattices used in this study was selected based on the data obtained from uniaxial unconfined compression testing of various 3D printed symmetric lattice-based structures fabricated using acrylonitrile butadiene styrene (ABS) thermoplastic filaments in an FDM-based 3D printer (UP Box). 3D cylindrical structures based on cubic or hexagonal lattices were developed for this study. Compression testing was performed according to the ASTM D695-10 testing standards protocol for rigid plastics using an Instron 1126 compression testing machine (Instron, Melbourne, Australia) and a crosshead speed of 1 mm s^−1^. The structures were compressed until failure and the compressive strength (at the point of fracture) and yield strength (stress at the beginning of plastic deformation) were calculated. Table S1 (Supplementary Materials) gives a compilation of the various CAD designs tested and the data obtained from uniaxial unconfined compression tests performed on 3D printed ABS-based structures developed using these CAD models. The design that imparted the highest mechanical strength to the 3D structure was selected as the design for use with chitosan hydrogel printing ink. It was assumed that since the property tested (compressive strength and yield strength) depends predominantly on the internal architecture of the structure (made of the same material), the trend obtained for ABS printed structures would reflect what would be obtained for the chitosan hydrogel-based equivalents [49,50]. Hence, a hollow cylinder enclosing cubic lattices, as shown in Figure 9, was used for all investigations undertaken with chitosan hydrogel printing inks.

The final selected CAD model for use with the chitosan hydrogels as the printing ink was a 15 mm diameter × 16 mm high cylindrical structure comprising of a 4 mm thick hollow cylinder enclosing ordered cubic lattices. The cubic lattice region is made of individual struts (0.7 mm thick) placed 1.5 mm apart within individual layers to obtain 1.5 mm × 1.5 mm macropores within the final printed structure. Overlying layers are rotated by 0°-90°-0° along the Z direction as the printing progresses (Figure 9). A plastic-based extrusion nozzle with 0.42 mm internal diameter was used to print the 3D structure.

3D printed chitosan hydrogel-based scaffolds 15 mm in diameter and 16 mm high (in the wet state) were comprised of 156 printed layers of hydrogel laminated to each other with appreciable adhesion, resulting in a stable 3D structure at room temperature. The 3D printed structures can be delaminated when in the hydrated state but when dehydrated via air-drying, the layers showed strong adhesion, thus restricting delamination.

### 4.4. 3D Printing

#### 4.4.1. Hydrogel Rheology 

The printing ink is prepared by dissolving 5% *w*/*v* chitosan in purified water in the presence of 2% *v*/*v* acetic acid under constant stirring for 12 h followed by centrifugation at 4000 rpm for 5 min.

The rheological properties of the chitosan hydrogel-based inks were studied using an AR2000 rheometer (TA Instruments, New Castle, DE, USA) with a cone-plate geometry (60 mm diameter, 2°0’37” cone angle, and 63 μm gap) at room temperature and pressure. The variation in the viscosity of freshly prepared 5% *w*/*v* chitosan hydrogel (in 2% *v*/*v* acetic acid solution; pH 2.8) with increasing shear rates was examined in stepped flow experiments under a step ramped shear rate from 0.01 to 100 s^−1^ at 25 °C. A strain sweep was performed at a frequency of 1 Hz for strains of 0.01% to 500% to identify the linear viscoelastic response of the hydrogels with respect to strain. A frequency sweep was performed at 2% strain under oscillating frequency varying from 0.01 Hz to 100 Hz. The actual shear rate experienced by chitosan hydrogel during extrusion can be estimated using the Hagen Poiseuille equation [51] modified with a Rabinowitsch correction as explained in [52]. The estimated shear rate at the tip of the 0.42 mm internal diameter nozzle when printing at a speed of 6 mm s^−1^ is 72 ± 7 s^−1^.

#### 4.4.2. Printing Process 

During the printing process, the layers of the printed hydrogel are laminated on to each other using 0.7–1.5% ethanolic sodium hydroxide solutions (70% *v*/*v* ethanolic NaOH). This is currently a manual process facilitated by using a modified G-code that ensures that the printing is paused, and the extrusion nozzle is lifted (along *z*-axis) away from the print platform while ethanolic NaOH solution is introduced. Before continuing with the printing process, the excess ethanolic NaOH is wiped off. Continuing printing without wiping off NaOH resulted in clogging of the printing nozzle. After printing, the 3D scaffolds were rested in 1 M NaOH solution for 24 h to ensure complete gelation of the hydrogel via the deprotonation of the amine groups in the chitosan hydrogel. The scaffolds are then washed thoroughly with purified water until neutral pH. Hydrated scaffolds were stored in purified water at room temperature until further use.

### 4.5. Mineralisation

The hydrated 3D chitosan hydrogel-based scaffolds were mineralised using the McGrath method as described elsewhere following some modifications [22]. Briefly, pH neutralized scaffolds are soaked alternately in CaCO_3_ precursor ion solutions for 24 h each. The precursor ion solutions used here were calcium chloride dihydrate and sodium hydrogen carbonate (CO_2_ bubbled at 3 L h^−1^ for 15 min). The concentration of the precursor ion solutions was varied from 0.1 M to 0.5 M. The scaffolds are then soaked in a saturated solution of CaCO_3_ (CO_2_ bubbled at 3 L h^−1^ for 15 min) for 7 days. The saturated solution of calcium carbonate (herein called Kitano), is prepared by dissolving 2.6 g of CaCO_3_ in 1 L of purified water under CO_2_ bubbling at 3 L h^−1^ for 8 h. The solution is filtered, and the filtrate bubbled with CO_2_ for a further 30 min at 3 L h^−1^.

Polyacrylic acid (PAA) is used as a crystal growth modifier and inhibitor in this study. For experiments carried out in the presence of PAA, 2.5% *w*/*w* PAA (with respect to the dry weight of the 3D chitosan scaffold) was added to each of the mineralisation solutions following CO_2_ bubbling. Further steps followed in the mineralisation process are the same as above.

### 4.6. Bulk Gelation and 3D Printing

To study the effect of the printed macropores and the layer-by-layer build of the 3D scaffold on the extent of mineralisation that could be achieved within the 3D structure, 3D scaffolds were fabricated as solid cylinders without macropores via bulk gelation and compared with those of 3D printed scaffolds with macropores. Solid cylinders were fabricated via bulk gelation of 15 mm diameter × 16 mm tall cylindrical columns of chitosan hydrogel that was gelled via slow deprotonation over a period of 7 days in the presence of 1 M NaOH. The process renders the hydrogel insoluble and hence assumes a cylindrical shape without any macropores. To compare, 15 mm diameter × 16 mm high 3D printed scaffolds were fabricated with 1.5 mm × 1.5 mm macropores. Both the 3D structures were mineralised via the McGrath method in the presence of 2.5% *w*/*w* PAA (with respect to the dry weight of each of the 3D scaffolds respectively). The EDS images of cross-sections of the resultant composites were compared to analyse the extent of mineralisation achieved throughout the 3D structure in each case.

### 4.7. Dehydration of As-Printed Scaffolds

The previously reported study on 2D chitosan thin films followed dehydration of the scaffolds prior to mineralisation. To validate this step, the as-printed chitosan hydrogel-based scaffolds (following gelation in 1 M NaOH) were dehydrated via three techniques and their effects on the porosity, structural integrity, mechanical resistance to deformation under compression and rehydration behaviour were compared. The technique that imparted the most favourable properties was used to dehydrate the final composite. The data obtained were also used to develop a preliminary understanding in identifying a singular technique that aided in increasing the mechanical strength of the 3D scaffolds.

(a) Air-drying: the scaffolds were subjected to air-drying under laminar flow conditions over a period of 24 h.
(b) Freeze-drying: the scaffolds were pre-frozen in liquid nitrogen after removing any excess solvent by simple pat drying and then freeze-dried using a VirTis Sentry freeze-drier (SP Industries, Warminster, PA, USA) for a period of 24 h.
(c) Critical point-drying: the scaffolds were equilibrated successively in increasingly concentrated ethanol solutions (30% to 95%) for 10 min each and then in absolute ethanol three times for 20 min each. Ethanol was then replaced by acetone via equilibration of the scaffold in acetone three times for 20 min each. Acetone is easily replaced with liquid CO_2_ during the drying process in the critical point dryer. The samples were dried using a Baltec CPD 030 critical point dryer (BAL-TEC GmbH, Balzers, Liechtenstein) at the 31 °C and 1072 psi, the critical point temperature and pressure of liquid CO_2_.

### 4.8. Swelling Behaviour 

To study the rehydration and swelling behaviour of dehydrated chitosan hydrogel-based scaffolds, air-dried, freeze-dried, or critical point-dried scaffolds were rehydrated in 1× PBS for 7 days. The swelling ratio and volume change in dried scaffolds were calculated based on the following equations:

(1) $$\mathrm{Swelling}\;\mathrm{ratio}\;=\frac{M_{w}}{M_{d}}$$

(2) $$\%\;\mathrm{Volume}\;\mathrm{change}=\frac{\left(V_{w}-V_{w}V_{d}\right)}{V_{d}}\;\times 100\%$$

where M_d_, M_w_, V_d_ and V_w_ are the mass and volume of the scaffold in the dried and rehydrated states respectively.

### 4.9. Characterisation

The 3D chitosan hydrogel-based scaffolds and composites were characterised by SEM, cryo-SEM, powder XRD, and EDS. For SEM, the dehydrated scaffolds were mounted on aluminium stubs using double-sided carbon tape and coated with 30 nm of platinum using a JOEL JFC-1500 ion sputtering device (JEOL, Akishima, Tokyo, Japan). The images were acquired using a JEOL JSM-6610LA field emission SEM (JEOL, Akishima, Tokyo, Japan) in secondary electron imaging (SEI) mode at an accelerating voltage of 20 kV. EDS images were acquired in backscattered electron image (BEI) mode at 20 kV accelerating voltage at a working distance of 10 mm. In order to visualise the mineralisation in between the printed layers of the 3D composite structure, mineralised scaffolds were delaminated before air-drying, thus exposing the features in between the layers of the scaffold.

Cryo-SEM was used to analyse the intrinsic porosity of hydrated and rehydrated chitosan hydrogel-based structures. Images were acquired on a JEOL 6500F SEM (JEOL, Akishima, Tokyo, Japan) equipped with a cryo-stage in SEI mode. Hydrated chitosan hydrogel-based scaffolds were frozen using a jet freezing system, wherein, a small piece of the samples was sandwiched between two Cu holders and transferred to the jet freezing chamber under a constant flow of propane gas. After pressure freezing the sample, it was dropped into a propane/liquid nitrogen mix and then transferred into the cryo-chamber within a cooled −120 °C high vacuum environment. Rehydrated samples, on the other hand, were frozen in liquid nitrogen prior to transferring to the cryo chamber. The samples were then sublimated for 10 min at −90 °C to expose the internal features of the sample, twice coated with platinum and transferred to the microscope viewing platform. The temperature of the microscope chamber was stabilised at −120 °C while imaging. Accelerating voltages of 3 kV and 5 kV respectively were used to image hydrated and rehydrated samples.

For powder XRD, the scaffolds were powdered in liquid nitrogen and tested using the PANalytical X’Pert Pro powder diffractometer (Malvern Panalytical, Westborough, MA, USA) using Cu-Kα radiation. The XRD patterns were obtained with generator settings of 45 kV and 40 mA; scans were acquired for 2θ values from 5° to 70°. The resulting diffraction patterns were compared with those of known compounds from the database, using the software X’Pert Highscore 2.2c (PANalytical Inc., Westborough, MA, USA).

## 5. Conclusions

The prospect of fabricating symmetrically porous 3D scaffolds using chitosan hydrogels as the printing ink in a custom-designed nozzle extrusion-based 3D printer was investigated. Structurally stable 3D scaffolds with macropores, micropores and nanopores were successfully fabricated using 5% *w*/*v* chitosan hydrogel as the printing ink. The structural stability of the scaffolds using different dehydration methods was investigated. Freeze-dried and critical point-dried scaffolds were found to retain the inherent porosity of the hydrogel (micro and nanopores) and displayed minimal dimensional shrinkage. However, their mechanical resistance to deformation was low. Air-drying was found to be a suitable dehydration method from the bone graft materials application point of view, despite the observed significant dimensional shrinkage. This is due to (1) their comparably higher mechanical resistance to deformation; (2) the observation that printed macropores are retained upon dehydration (albeit with some dimensional shrinkage); and (3) the prospect of regaining some of the porosity originally observed in the as-printed scaffolds (nano and micropores) upon rehydration.

The applicability of the McGrath mineralisation method in developing 3D chitosan-calcium carbonate composites was investigated. The as-printed chitosan hydrogel-based scaffolds were mineralised *via* the McGrath method in the presence and absence of PAA as the crystal growth modifier. The printed macropores and the layer-by-layer build of the 3D chitosan scaffolds increased the extent of mineralisation achieved in the final composite. By using 2.5% *w*/*w* PAA and modifying the McGrath mineralisation process to perform the mineralisation when the chitosan hydrogel scaffold is in its hydrated state, pancake-like calcium carbonate formation, which is in intimate association with the hydrogel matrix, as obtained previously on 2D thin films, is replicated with 3D chitosan hydrogel-based scaffolds. However, more research is required to obtain an optimal distribution of this particular morphology throughout the 3D printed scaffold.

## Figures and Tables

**Table 1:** Swelling behaviour of chitosan hydrogel-based scaffolds dehydrated via different methods after soaking in 1× phosphate buffered saline (PBS) for one week. The solution was not changed during the experiment.

Reswelling Behaviour in 1X PBS	Air-Drying	Freeze-Drying	Critical Point-Drying
Solvent uptake ratio	2.1 ± 0.2	5.2 ± 1.0	5.5 ± 0.4
% Volume change	75.4 ± 12.0	No change	No change

## Supplementary Materials

The following are available online at http://www.mdpi.com/2079-4983/10/1/12/s1, Figure S1: The custom-designed 3D printer used in this study, Table S1: The variation in yield stress and compressive strength of the 3D printed ABS-based structures with different architectures.
