# Supplementary material for: Towards the Development of Artificial Bone Grafts: Combining Synthetic Biomineralisation with 3D Printing
Source: J Funct Biomater. 2019 Feb 20;10(1):12. doi: 10.3390/jfb10010012 (PMC6462944; doi:10.3390/jfb10010012)
Supplement: Supplementary file 1 [file jfb-10-00012-s001.pdf]

Supplementary Materials: The 3D printer (Figure S1) is equipped with additional functionalities including a heat gun (room temperature – 90 °C) allowing temperature-dependent layer lamination in addition to the physical crosslinking-based lamination technique used in this study. The printer uses the RAMBO microcontroller developed by Ultimaker based on the open-source Arduino Mega 2560 platform paired with an open-source Slic3r and Repetier Host 3D model slicing.

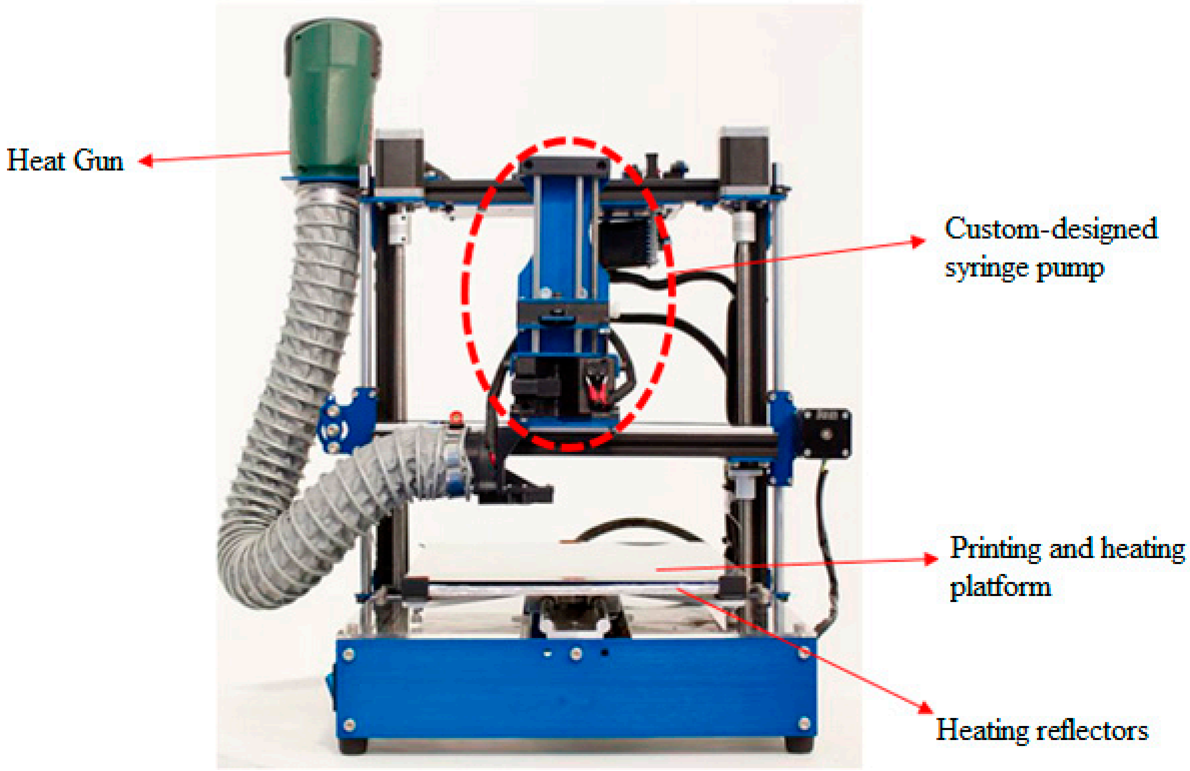

Figure S1: The custom-designed 3D printer used in this study.

1.1 Uniaxial compression testing of 3D printed ABS-based structures

Table S1 is a compilation of the various CAD designs tested and the data obtained from uniaxial unconfined compression tests performed on 3D printed acrylonitrile butadiene styrene (ABS) thermoplastic plastic-based structures developed using these CAD models. All the structures tested were 20 mm (diameter) × 40 mm (height), the thickness of individual struts forming the 3D structure is 0.9 mm. The lattices were generated by using a basic structure in which the first layer contains struts placed 1.5 mm apart from each other and following specific layer rotations patterns for the subsequent layers as given in Table S1. Compression testing was performed by ASTM D695-10 testing standards protocol for rigid plastics using Instron 1126 compression testing machine at a crosshead speed of 1 mm/s. The structures were compressed until failure and the compressive strength (at the pint of fracture) and yield strength (stress at the beginning of plastic deformation) were calculated.

Table S1: The variation in yield stress and compressive strength of the 3D printed ABS-based structures with different architectures.

**Geometry of the test specimens**	**CAD Model**	**Yield stress (MPa)**	**Compressive strength (MPa)**
Cylinder based cubic lattice<br><br>(layer rotation 0-90-0°)	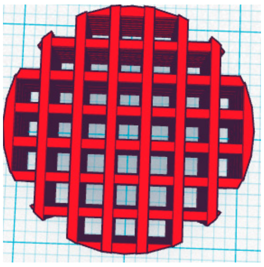	2.44 ± 0.01	3.00 ± 0.01
Cylinder based hexagonal lattice<br><br>(layer rotation 0-60-120°)	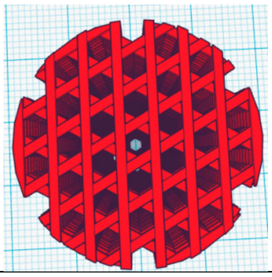	0.76 ± 0.01	2.73 ± 0.01
Hollow cylinder<br><br>(1 mm wall thickness)	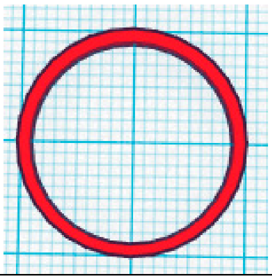	35.22 ± 0.02	46.92 ± 0.02
Hollow cylinder<br><br>(2 mm wall thickness)	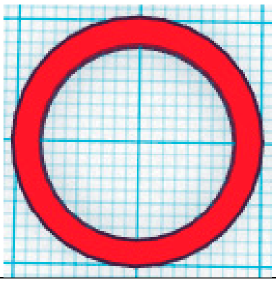	30.70 ± 0.01	40.51 ± 0.01
Hollow cylinder<br><br>(5 mm wall thickness)	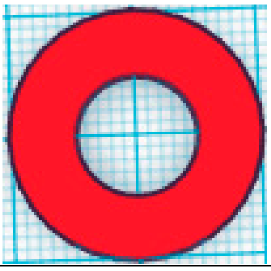	22.31 ± 0.01	31.20 ± 0.02

Hollow cylinder (4 mm wall thickness) enclosing cubic lattices	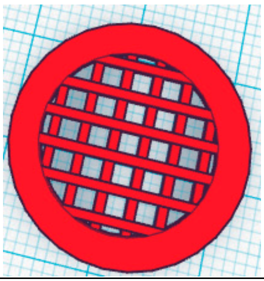	34.51 ± 0.01	38.91 ± 0.02
Hollow cylinder (4 mm wall thickness) enclosing hexagonal lattice	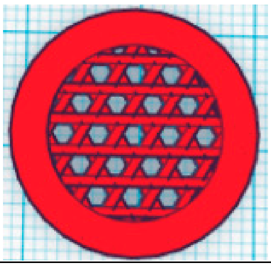	31.42 ± 0.01	34.28 ± 0.01

Data show that cubic lattices (0-90-0° rotation of the overlying printed layers) offered better resistance to complete fracture compared to hexagonal lattices (0-60-120° rotation of overlying layers). This could be due to variation in the deformation mechanisms occurring in each of the designs. Li *et al*. explains that the variation in the unit cell architecture of repetitive lattice-based structures can cause either bending or buckling deformation under compression which in turn affects the resultant modulus, compressive strength and plasticity of the entire structure. Cubic lattices were found to show buckling deformation which resulted in a higher modulus and compressive strength compared to rhombic dodecahedron lattices which showed bending deformation at the nodes [1]. A hexagonal lattice is comparable to a rhombic dodecahedron lattice with regard to the fibre laydown pattern and hence most likely bending deformation at the nodes occurs. Buckling deformation causes better fracture resistance compared to the deformations occurring in hexagonal lattices. Furthermore, the data also showed that hollow cylinders with thinner walls are more stable under compression. This could be attributed to the higher second moment of area of thinner-walled hollow cylinders, resulting in a higher force required to initiate buckling fracture in the cylinder [2].

Based on these data, it was deduced that a structure with a hollow cylinder enclosing ordered cubic lattices may provide a higher resistance to fracture compared to the models based on hollow cylinders and symmetric cubic lattice structures individually. This assumption was confirmed by the data obtained for ABS-based 3D printed structures based on a combination of hollow cylinders and cubic lattices as shown in Table S1. Based on this initial work using ABS filament-based 3D printed structures, the CAD model depicted in Figure 8 of the manuscript was used as the sole model for all further research. The thickness of the hollow cylinder was chosen as 4 mm such that a stable scaffold could be fabricated using hydrogels as the ink.

1. Levengood, S.K.L.; Zhang, M. Chitosan-based scaffolds for bone tissue engineering. *Journal of Materials Chemistry B* **2014**, *2*, 3161-3184.
2. Griffin, D. *Inelastic and creep buckling of circular cylinder due to axial compression, bending, and twisting;* Westinghouse Electric Corp., Pittsburgh, Pa.(USA). Advanced Reactors Div.: 1973.
